# Mitigating the risk of inflammatory type primary graft dysfunction by applying an integrated approach to assess, modify and match risk factors in lung transplantation

**DOI:** 10.3389/frtra.2024.1422088

**Published:** 2024-08-20

**Authors:** Sue A. Braithwaite, Elize M. Berg, Linda M. de Heer, Jitte Jennekens, Arne Neyrinck, Elise van Hooijdonk, Bart Luijk, Wolfgang F. F. A. Buhre, Niels P. van der Kaaij

**Affiliations:** ^1^ Department of Anesthesiology, University Medical Center Utrecht, Utrecht, Netherlands; ^2^ Department of Pulmonology, University Medical Center Utrecht, Utrecht, Netherlands; ^3^ Department of Cardiothoracic Surgery, University Medical Center Utrecht, Utrecht, Netherlands; ^4^ Department of Anesthesiology, University Hospitals Leuven, Leuven, Belgium

**Keywords:** lung transplantation, primary graft dysfunction, PGD risk matching, immunomodulation, EVLP, extended EVLP, donor lung allocation

## Abstract

Long-term outcome following lung transplantation remains one of the poorest of all solid organ transplants with a 1- and 5-year survival of 85% and 59% respectively for adult lung transplant recipients and with 50% of patients developing chronic lung allograft dysfunction (CLAD) in the first 5 years following transplant. Reducing the risk of inflammatory type primary graft dysfunction (PGD) is vital for improving both short-term survival following lung transplantation and long-term outcome due to the association of early inflammatory-mediated damage to the allograft and the risk of CLAD. PGD has a multifactorial aetiology and high-grade inflammatory-type PGD is the result of cumulative insults that may be incurred in one or more of the three variables of the transplantation continuum: the donor lungs, the recipient and intraoperative process. We set out a conceptual framework which uses a fully integrated approach to this transplant continuum to attempt to identify and, where possible, modify specific donor, recipient and intraoperative PGD risk with the goal of reducing inflammatory-type PGD risk for an individual recipient. We also consider the concept and risk-benefit of matching lung allografts and recipients on the basis of donor and recipient PGD-risk compatibility. The use of ex vivo lung perfusion (EVLP) and the extended preservation of lung allografts on EVLP will be explored as safe, non-injurious EVLP may enable extensive inflammatory testing of specific donor lungs and has the potential to provide a platform for targeted therapeutic interventions on lung allografts.

## Introduction

Outcome following lung transplantation remains the poorest of all solid organ transplants (1) with 1- and 5-year survival 85% and 59% respectively for adult lung transplant recipients transplanted in the era since 2010 (2). Survival following lung transplantation is improving according to the modernity of the era of transplantation, with the median survival following transplantation during the era 2010 onwards 6.7 years compared to median survival of 4.7 years following transplantation during the era 1992–2001 (2). In contrast, the incidence of chronic lung allograft dysfunction (CLAD) remains unchanged, with 50% of patients developing CLAD in the first 5 years following transplantation (3). Prognosis following the identification of CLAD depends on the phenotype of the allograft dysfunction (4), with mean survival following diagnosis of the more common bronchiolitis obliterans syndrome (BOS) 3–5 years and following diagnosis of restrictive allograft syndrome (RAS) 1–2 years (3). Other factors limiting long-term survival include side effects of immune suppression therapy, such as infections, renal failure and solid organ cancer.

Short-term outcome of lung transplantation is highly dependent on the occurrence and eventual extent of primary graft dysfunction (PGD) in the lung allograft in the acute post-operative phase. A prospective multicenter cohort study of over 1,500 patients showed that PGD following lung transplantation is significantly associated with increased overall mortality, prolonged mechanical ventilation and prolonged length of stay (5). The same study demonstrated that recipients who develop PGD lose approximately a month of life at one year and around 5 months at 3 years compared to recipients who do not develop PGD. PGD occurs after approximately 30% of lung transplantations and the incidence of high grade PGD at 48 and 72 h is 15%–20% (6). PGD can therefore be regarded as a commonly reported and highly significant early complication following lung transplant with negative consequences for both early and delayed survival. High grade PGD is also a risk factor for developing CLAD, independent of other CLAD-associated risk factors such as acute rejection and community acquired viral infections (7).

Many donor-, recipient- and intraoperative risk factors for the development of PGD have been identified but as yet an integrated and individualized approach to PGD risk identification and modification throughout the full transplantation donor-recipient-intraoperative continuum remains elusive. At present the known donor, recipient and intraoperative risk factors are regarded as mainly epidemiologically-derived dichotomous inputs for high or low PGD risk. In the following article we aim to show that the PGD risk conferred by an individual lung transplant continuum may potentially be derived from objective diagnostic parameters based on the known aetiology of PGD and the concept of lung ischemia reperfusion injury (LIRI). We begin by reviewing the inflammatory processes which contribute to the development of PGD in donor lung allografts. Subsequently, the role of the immunophenotype and inflammatory status of a specific recipient in developing PGD is described. Intraoperative PGD-associated risk factors and their possible lung-inflammatory aetiology are also reviewed and the cumulative effect of inflammatory risk in all three variables on the outcome of a specific transplant continuum is explored. On the basis of this, we conceptualize a model examining the feasibility of PGD risk compatibility-matching and targeted risk modification of specific donor lung allografts in individual recipients.

Extended preservation of lung allografts on ex vivo lung perfusion (EVLP) gives future perspectives to this model by providing both the opportunity to assess the real-time inflammatory status of specific lung allografts and the time to contextualize the eventual donor allograft PGD risk in any specific individual transplant continuum.

### Primary graft dysfunction: definition and aetiology

Primary graft dysfunction describes the functional and morphological sequalae of lung allograft injury incurred in the acute transplant phase and is diagnosed and graded according to the 2016 International Society for Heart and Lung Transplantation Primary Graft Dysfunction Definition at four time points, every 24 h, up to the first 72 h after reperfusion of the second donor lung in the recipient (8) (Table 1). The injury present in the lung allograft represented by the diagnosis and grade of PGD may be incurred at any point during the full transplantation continuum from the ambulant and/or peri-mortal donor phase, the period of procurement, the allograft preservation phase and both during and following reperfusion in the recipient. Indeed, a number of epidemiologically-derived donor-, recipient- and intraoperative-related risk factors for the development of PGD are well known and suggest potential aetiological causes of this injury (6, 9). Donor factors are related to donor smoking history, extremes of donor age and processes in the donor lungs prior to procurement including (micro-) aspiration or contusion (10). Recipient risk is primarily related to the primary diagnosis with pulmonary hypertension, pulmonary fibrosis and sarcoidosis conferring the highest risk of PGD but with recipient obesity also conferring additional PGD risk (11, 12). Intraoperative risk factors are known to be related to the use of extra corporeal circulation during the procedure and the transfusion of 4 or more units of red blood cells (6). The multifactorial nature of the aetiology of lung injury causing PGD means that there is at present no specific treatment once the diagnosis of PGD has been made: supportive care is best-practice with consideration given to lung-protective ventilation strategies and if necessary in high-grade PGD, the initiation of extra corporeal membrane oxygenation (ECMO) to facilitate lung-rest (13).

**Table 1:** PGD grade according to the 2016 international society for heart and lung transplantation primary graft dysfunction deﬁnition (8).

Grade	Pulmonary oedema on chest x-ray	PaO_2_/FiO_2_ ratio
PGD grade 0	No	Any
PGD grade 1	Yes	>300
PGD grade 2	Yes	200–300
PGD grade 3	Yes	<200

PaO_2_/FiO_2_ ratio: ratio of the partial pressure of oxygen in arterial blood (PaO_2_) at a measured fraction of inspired oxygen (FiO_2_). PaO_2_/FiO_2_ should ideally be measured at a positive end-expiratory pressure of 5 cmH_2_O with an FiO_2_ of 1.0 while patients are on mechanical ventilation. At a lower FiO2 the PaO2 may be overestimated with regard to the extent of lung injury due to the effect of hypoxic vasoconstriction in the lungs. The use of extracorporeal life support (ECLS) with bilateral pulmonary oedema on the chest x-ray should be graded as grade 3 PGD. The use of ECLS for non-hypoxic indications without pulmonary oedema on chest x-ray imaging should be considered ungradable and explicitly recorded separately.

Two different phenotypes of high grade PGD have been described and their clinical course within the first 72 h following reperfusion has been identified (14). One form of PGD results in a transient allograft dysfunction that resolves within the first 72 h post-transplant and is thought to be caused by hydrostatic alveolar oedema secondary to causes such as perioperative hyperperfusion of the donor lung (15). The more severe form of PGD is expressed as persistent lung dysfunction that either attenuates within the first 48 h following transplantation or remains persistent at 72 h and is associated with an increased mortality (14, 16). This form may be considered as an inflammatory phenotype of PGD. A mixed phenotype is also observed, reflecting the complex array of aetiological factors driving high grade PGD.

The cause of lung injury in PGD is multiphasic (17) with the extent of lung injury being dependent on the specific immunophenotype of the recipient which regulates the acute inflammatory response following reperfusion. In order to explore this concept, the aetiology of lung ischemia-reperfusion injury (LIRI) will be reviewed.

### Lung ischemia-reperfusion injury

Lung allografts are procured in a semi-inflated state ensuring an intra-alveolar source of oxygen (in recruited lung parenchyma) during the phase of static cold storage. The vascular endothelium, however, experiences relative hypoxia and in combination with the lack of mechanotransduction caused by absent blood flow through the capillaries, macrophages and other immune cells are induced to produce reactive oxygen species (ROS) (17). Nicotinamide adenine dinucleotide phosphate (NADPH) oxidase, which is present in endothelial cells, is one of the major sources of ROS which in turn activates nitric oxide synthases (NOS), nuclear factor-kappa B and proinflammatory cytokines, causing an upregulation of cell-surface adhesion molecules in the endothelium (18). ROS also has a role in causing mitochondrial damage by its injurious effects on the integrity of the outer mitochondrial membrane which in turn triggers cell apoptosis (17). Hypoxia itself also causes endothelial cell mitochondrial damage through ATP depletion resulting in a decrease in the membrane potential of the mitochondria. This leads to mitochondrial calcium loss which is also a potent driver of cell apoptosis. Upon reperfusion in the recipient, the upregulated cell-surface adhesion molecules, proinflammatory cytokines and damaged cells expressing damage associated molecular patterns (DAMPs) cause infiltration of the vascular endothelium with recipient macrophages, neutrophils and other cells of the innate immune response (18).

Macrophages recognizing DAMPs with pattern recognition receptors (PRR) such as toll like receptors (TLRs) and responding to cytokines, become activated and exacerbate the inflammatory injury to the endothelium (19). DAMPs have a role in amplifying inflammation by binding to PRRs and are not only relevant in LIRI but also the amplification of any existing inflammation present in the donor lung after reperfusion.

Insults incurred by the donor lung before procurement, due to a number of causes such as contusion, (micro-) aspiration, ventilator-associated lung injury (VILI) and infection, may result in injury to the alveolar epithelium. Damage to type I alveolar cells and disruption of the basement membrane of the alveolus leads to a breakdown of the integrity of the endothelial-epithelial barrier and has the potential to induce an influx of fluid from the endothelium into the alveolus upon reperfusion of the lung in the recipient. The receptor for advanced glycated end products (RAGE) is a PRR which is localized in the basolateral membrane of alveolar type I cells and binds to DAMPs such as the type high mobility group box 1 proteins (HMGB 1) further inducing inflammation via pathways such as natural killer cell mediated interleukin (IL) 17 expression and neutrophil activation (20). Elevated plasma levels of RAGE after reperfusion of lung allografts are associated with the occurrence of PGD (21). The binding of RAGE to specific DAMPs leads to the sequestration of neutrophils, facilitating their migration through the disrupted endothelial-epithelial barrier into the alveolus. Activated neutrophils subsequently secrete leukotrienes, oxidases and other pro-inflammatory molecules exacerbating damage to the type I alveolar cells and disrupting the function of the surfactant-producing type II alveolar cells. Alveolar macrophages also play a prominent role by secreting pro-inflammatory cytokines such as IL-1β, IL-6, -8, interferon-γ and tumour necrosis factor alpha (TNF-α) (9). Another marker of epithelial injury is collagen type V (Col-V), a collagen type normally sequestered within fibrils of type I collagen, the major collagen in the lung. Col-V becomes exposed by the epithelial disruption and subsequently becomes a powerful autoantigen and driver of the inflammatory mediated damage in PGD (22).

### The role of the recipient immunophenotype and other recipient-related inflammatory risk factors for PGD

The specific immunophenotype of the recipient has a leading role in the development of inflammatory-type PGD following lung transplantation (23–26). The recipient's individual acute immune response and therefore the degree of inflammation triggered by LIRI is dependent on the balance of pro- and anti-inflammatory signaling, which is in turn dependent on the specific immunophenotype of the recipient.

A number of inherited immunophenotypes are known to have an association with increased PGD risk. Certain genetic variants of IL-17 receptors (IL-17R) in lung transplant recipients are an example. The presence of an IL-17R PGD risk-genotype leads to an up-regulation in the proliferation of IL-17 T-cells which in turn initiates a pro-inflammatory positive feedback loop with production of inflammatory cytokines and growth factors, such as IL-1β, IL-6 and IL-23 and subsequent inflammatory cell infiltration in the lung allograft following reperfusion (23). In lung allografts with additional existing damage to the endothelial-epithelial barrier due to donor-related causes this immunophenotype-dependent amplified inflammatory response may have the potential to significantly worsen lung injury. Other known genetic variations causing an up-regulated pro-inflammatory response and an increased risk of PGD include variations in the innate immune mediator long pentraxin-3 (PTX3) (26). PTX3 is produced at sites of inflammation by antigen presenting cells of the innate immune system as a result of pro-inflammatory IL-1 and toll like receptor (TLR) 4 signaling pathways and is found to be elevated secondary to a range of inflammatory and ischemic conditions (27).

In addition to the increased risk of PGD conferred by specific recipient pro-inflammatory immunophenotypes, genetic variants leading to the sub-optimal function of anti-inflammatory processes in the recipient also play an important role in the lung injury associated with PGD. In one of the first major studies investigating the association of inflammation-associated genetic variations in recipients and PGD risk it was found that variations in 2 genes of the prostaglandin E2 (PGE2) family carry an increased PGD risk (25). One of the PGD-risk variants was in PTGER4 which plays an important role in regulatory T cell (Treg) function: the risk variant led to reduced Treg function and the implication that an impaired anti-inflammatory response increases PGD risk in these recipients. Furthermore, a study from the same group found that decreased Treg function plays an important role in the increased PGD risk observed in a cohort of obese patients who display a general systemic pro-inflammatory phenotype (24). Such individuals have been found to have acquired increased levels of IL-18 leading to the Treg functional suppression. The same study also showed in a murine PGD model that inhibition of IL-18 mitigates lung inflammation.

Broadening this concept away from an inherited immunophenotype but with respect to the acquired immune response, certain lung tissue-restricted antigens may be exposed in specific recipients in the pre-transplant phase, such as collagen type-V (Col-V) and -I (Col-I) in addition to Kα1 tubulin (Kα1 T). The presence of these self-antigens (SAgs) has been demonstrated in recipients pre-transplant with idiopathic pulmonary fibrosis (IPF) patients and cystic fibrosis patients demonstrating the highest prevalence (28). Pre-existing antibodies to SAgs in lung transplant recipients is strongly associated with the development of high grade PGD (22, 28, 29) and are also a significant risk factor for the development of bronchiolitis obliterans syndrome in the chronic phase post-transplant (28, 29).

A further relevant pre-transplant molecule of note with a strong association with PGD is cell-free hemoglobin (CFH) (30) which accumulates due to red blood cell (RBC) damage. Conditions with a known association with increased pre-transplant CFH levels include sepsis, pulmonary hypertension (31) and pre-transplant extra corporeal life support (32). CFH is a powerful proinflammatory oxidant and when free in the circulation can be oxidized to drive oxidative-mediated damage of proteins and lipids. In the lung, elevated levels of CFH are associated with epithelial cell injury and increased permeability of the endothelium causing alveolar oedema.

### The innate immune response and role of the neutrophil in PGD

Multiple studies, in both clinical settings and in animal models, have shown that ischemia-reperfusion injury is associated with neutrophilic infiltration of lung allografts (33). Drivers of this are numerous molecular and cellular chemo-attractant pathways, including necroptotic cell death upon reperfusion (34). Sequestered donor neutrophils adherent to the capillaries and tissue resident macrophages in the donor lung also play an important role in the propagation of inflammation and the infiltration of the donor lung by recipient neutrophils (35). Neutrophils effect their inflammatory-mediated tissue damage through a number of processes including the amplification of inflammatory process, the excretion of injurious substances via granulocytes and by forming neutrophil extracellular traps (NETs) which are networks of extracellular fibers composed mainly of neutrophil DNA (36).

Pre-operative inflammatory status of the recipient as demonstrated by an elevated neutrophil to lymphocyte ratio has been shown to be associated with post-transplant graft failure and poor 3-year survival following lung transplant (37). Biomarkers associated with the formation of NETs (NETosis), such as IL-8, myeloperoxidase, and myeloperoxidase-DNA complexes may become raised perioperatively and up to 72 h post-operatively and have been shown to be strongly associated with the onset of high grade PGD in a clinical study (38). The role of NETs in PGD is multi-faceted due to their adherence to the lung endothelium and ability to provide a micro-network enabling the binding of platelets and inflammatory cells thus causing their activation and the propagation of inflammation. Furthermore, NETs may also facilitate microvascular thrombotic occlusion in the capillaries surrounding the alveoli.

### Intraoperative risk factors for PGD

Intraoperative risk factors for PGD include the use of extracorporeal circulation (ECC) as hemodynamic and/or ventilatory support during transplant. A large international multicenter analysis showed that lung transplants performed without ECC support had a lower PGD risk than if ECC was used (39). If ECC was required perioperatively, the use of perioperative ECMO/ECLS (Extracorporeal Life Support, an alternative term for ECMO) was associated with less PGD risk than cardiopulmonary bypass (CPB). This finding is corroborated by another study of a cohort of 55 patients showing PGD grade 3 at 72 h in 60% of patients supported perioperatively with CPB, 40% of patients supported with ECMO and in 15% of patients requiring no ECC support during lung transplantation (40). The same study also demonstrated that the use of CPB was associated with endothelial damage, a marker of which (Syndecan-1) was also predictive of the development of high grade PGD and other organ dysfunction. The aetiology driving the additional PGD risk conferred by ECC has not been fully explored, however the role of ECC-driven hemolysis and inflammation must be taken into consideration. A recent meta-analysis showed that mini- ECC systems (to which ECLS may be compared) produce significantly less hemolysis in comparison to CPB. Furthermore, the use of cardiotomy suction and active venous return in CPB (also a feature of ECLS support) also significantly increase cell free hemoglobin (41), which is, as previously explored, a potent driver of inflammation in the lung allograft. The degree of hemolysis caused by ECC may therefore have a role in both endothelial damage and PGD risk.

Other known intraoperative risk factors for PGD reflect an increased risk of a technically difficult surgical procedure and include perioperative transfusion of 4 or more units of packed red blood cells (42), allograft total ischemia times (43, 44) and prior cardiothoracic surgery (45). Transfusion of red blood cells has the potential to increase cell-free hemoglobin levels, which may play a role in the aetiology of the associated PGD risk.

The association of the recipient's immunophenotype and inflammatory status on intraoperative systemic inflammation during lung transplantation has not been studied. Intraoperative systemic inflammation is typically observed as a vasoplegic syndrome with high vasopressor requirements necessitating large volumes of intravascular fluids. High volumes of intravascular fluids are in turn a risk for PGD (46). A single center study showed that recipients with post-operative vasoplegia had worse short-term outcome, including duration of mechanical ventilation and ICU length-of-stay (47).

It is important to note that “best-practice” intra-operative management involving optimal ventilation strategies, fluid management and surgical technique has a vital role in mitigating for intraoperative PGD risk as injurious ventilation strategies with a high (reperfusion) FiO_2_, fluid overload and prolonged warm ischemia times for the lung allografts during implantation are all associated with increased incidences of PGD (46).

### Assessing and identifying recipient PGD risk based on a specific transplantation continuum

On the basis of the above, inflammatory-type PGD can be regarded as the morphological and functional outcome of injurious processes of multifactorial origin to which the donor lung is exposed during the transplantation continuum. Furthermore, the extent of injury expressed in the lung allograft may be dependent on the pro- and anti-inflammatory balance of the innate immune system of the individual recipient.

Individual PGD risk may therefore be theoretically contextualized on the basis and interaction of the three variables of the transplant continuum: the presence and extent of any epithelial and/or endothelial damage or inflammation present in the donor lung, the inflammatory status and immunophenotype of the recipient and risk of any known intraoperative factors which may lead to the initiation or exacerbation of lung injury. By identifying and assessing lung injury and/or inflammation and/or risk as an interaction of these three variables, eventual strategies may be developed to modify an individual recipient's PGD risk for a specific transplant continuum.

### Assessing allograft inflammatory risk

At present, the standard assessment of donor lungs is functional and morphological. The ratio of the partial pressure of oxygen (PaO_2_) in arterial blood at an inspired oxygen fraction (FiO_2_) of 100% (P/F ratio) is measured in the donor and any morphological abnormalities are diagnosed on a standard chest x-ray or chest CT. If the P/F ratio is above 300 mmHg and the imaging is normal the lungs are procured (48). If the P/F ratio is below 300 mmHg and/or there are abnormalities on the chest x-ray or CT, the lungs are then seen as marginal and may then undergo ex vivo lung perfusion (EVLP) with the aim of assessing their function before eventual transplant. During EVLP the lung allografts are perfused, rewarmed and ventilated once normothermia has been reached. Assessment of the lungs on EVLP is again functional, by following the P/F ratio, pulmonary vascular resistance and lung compliance, in addition to assessing eventual weight gain of the lungs as a proxy for the presence of extravascular lung water. Indeed, a composite scoring system of lung allografts on EVLP has been developed, the COMPLETE score, which takes into account functional and morphological parameters including lung weight, ultrasound evaluation, lactate level, the P/F ratio and compliance. This score shows correlation with short-term outcome following the transplant of EVLP allografts (49). Other advanced testing of the lungs includes ultrasound assessment of individual zones of the lung parenchyma for extravascular lung water (the CLUE score) (50, 51).

Diagnostic assessment of lung inflammation or epithelial and/or endothelial injury on EVLP is not routine and the lung function and morphology (weight and parenchymal oedema) serve as the only way to derive if there is lung injury or inflammation presenting as increased endothelial-epithelial (alveolar) permeability. In order to challenge this, an *ex vivo* lung perfusion score (Toronto Lung Score) has been developed (52) which utilizes the protein levels of interleukin-6 (IL-6) and IL-8 in the perfusate in combination with a functional assessment of oxygenation of the lung. This score has been validated and can predict which lung allografts on EVLP give good recipient outcomes (ventilator time post-transplant of less than 3 days). The length of time required to perform the cytokines assays limits their clinical utilization as it is not possible to use the results to aid clinical decision making during a standard EVLP run of 6 or less hours. A recent pilot study however validated a rapid perfusate diagnostic platform which is able to measure lung biomarkers including IL-6 and IL-8 within 45 min (53) meaning that clinically relevant inflammatory diagnostics of lung allografts is within reach.

Future perspectives in assessing eventual allograft injury and/or inflammation utilizing EVLP may include diagnostics aimed at specifying the aetiological cause of any inflammation diagnosed by cytokine assays as increased pro-inflammatory cytokines are still a non-specific marker of inflammation. In an ideal model the presence and degree of specific allograft epithelial and/or endothelial damage can be elicited and the activation of tissue-resident macrophages, endothelial adherent neutrophils and degree of NETosis determined. This may include determining epithelial injury markers in the perfusate and/or by bronchoalveolar lavage (BAL), such as Col-V and RAGE. BAL also gives the opportunity to investigate markers for NETosis, such as neutrophil elastase DNA (34). Endothelial cell integrity may also be considered and could be demonstrated by novel assays including detecting circulating cell free mitochondrial DNA in the EVLP perfusate (54). Such detailed aetiological inflammatory profiling of donor lungs would not only enable the application of targeted therapies in the future but would also enable a detailed PGD risk assessment by taking into account other PGD inflammatory risk variables in the specific transplant continuum such as those associated with the recipient and intraoperative process.

### Assessing recipient inflammatory risk

At present, inflammatory profiling of the recipient prior to lung transplantation is not standard practice. However, such inflammatory profiling both in the (semi-) elective waiting list phase and in the acute pre-operative (transplant) setting would give a detailed aetiological and inflammation-based estimation of PGD risk. During the waiting list phase, the immunophenotype of the recipient could be determined, and the presence of any known inherited immunophenotypes known to have an association with increased PGD risk elicited. The pre-operative (pre-transplant) real-time inflammatory status of the recipient may also be the target of diagnostic profiling. This would ideally include the presence of acquired humoral immunity to lung-specific SAgs and determination of the neutrophil to lymphocyte ratio (37). Furthermore, the pre-transplant presence of other molecular risk factors or aetiological causes for PGD, such as CFH, may be determined. By using such detailed pre-operative diagnostics of the recipient to provide a pre-transplant inflammatory profile or fingerprint, a more aetiologically based recipient-specific risk profile for high-grade PGD may be constructed.

### Assessing a recipient's specific intra-operative risk

An assessment of intra-operative inflammatory PGD risk for a specific individual would be possible by taking into account the following: risk factors for the use of ECC perioperatively, the likelihood of a complex procedure and risk of hemodynamic instability. A 3-point score to predict the necessity for unplanned ECC during lung transplantation has been developed, which assigns two points if the preoperative mean pulmonary artery pressure is greater or equal to 35 mmHg and one point for a lung allocation score (LAS) of greater or equal to 50 (55). Estimation of the surgical complexity of the transplantation may be made on the basis of risk of perioperative blood loss (56) [dependent on factors such as prior thoracic surgery, chronic (pleural) infection and the presence of major abnormal bronchial arteries]. More specific and real-time assays reflecting ongoing perioperative inflammation in the recipient have yet to be developed or utilized in a clinical setting but may in the future include perioperative assessments such as rapid cytokine assays, detection of plasma RAGE and rapid detection of CFH.

### The concept of appropriately matching recipient, donor and intra-operative inflammatory risk

The influence of risk matching donor lung allografts and recipients on post-lung transplant survival is well known, By categorizing high risk donor lungs on the basis of donor smoking history and extreme age and the recipient risk by a high LAS score it can be shown that matching high-risk donor lungs in high-risk recipients leads to worse outcomes than low-risk donor lungs in lower risk recipients (57). Taking this concept further by identifying donor-recipient compatibility on the basis of actual inflammatory PGD risk would enable the risk to be objectified for an individual recipient's transplant continuum and introduces the possibility of targeted mitigation of PGD risk.

**Figure 1:**
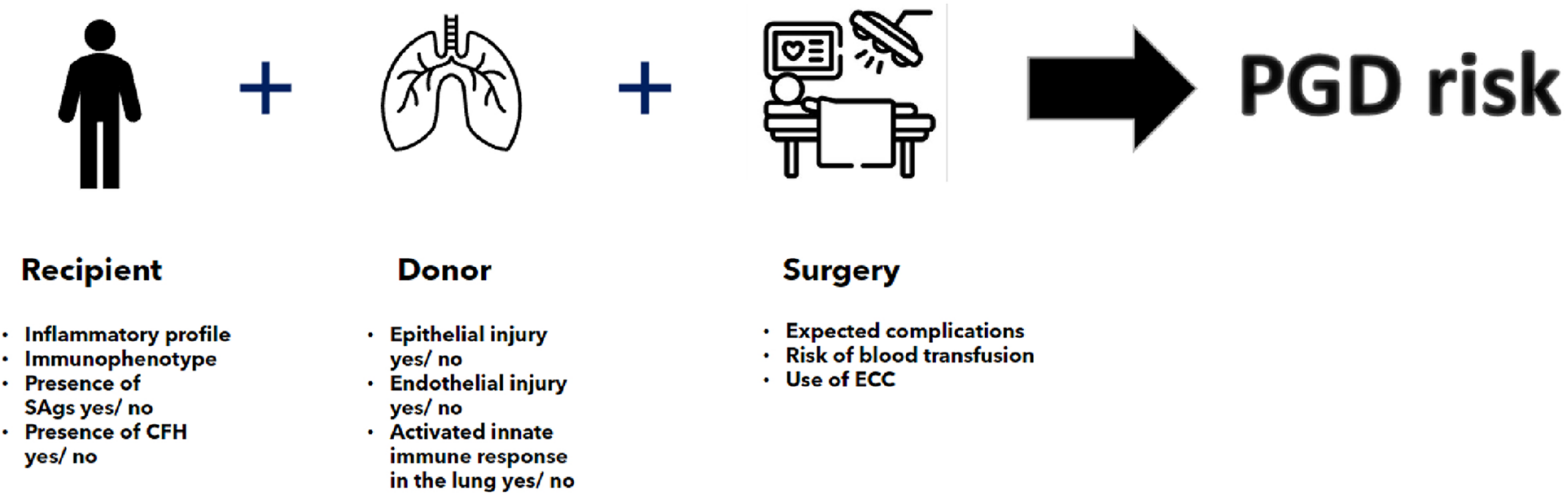
Individualized PGD risk estimation on the basis of recipient, donor and surgical risk factors as taken in the contex of a specific transplant continuum. SAg, lung-specific self-antigens; CFH, cell free haemoglobin; ECC, ectracorporeal circulation.

As we have seen, a number of inflammation-related risk factors are known which predispose a specific lung transplant recipient to PGD. In addition, a number of factors may predispose a specific lung allograft to an increased risk of high-grade LIRI (Figure 1). Conceptually, therefore, a lung transplant recipient with a high risk of PGD as based on their immunophenotype and pre-transplant inflammatory status will have a higher risk of severe PGD if they receive lung allografts with an inherent risk of LIRI. This risk may be compounded and exacerbated by an operative process conferring extra inflammatory insults. Such a recipient would therefore conceptually benefit from receiving lung allografts with a low risk of LIRI. Taking this concept further, a recipient with a low-risk immunophenotype and little or no pre-transplant inflammation, with a low intra-operative risk would be a more suitable recipient of lung allografts with a high risk of LIRI as the compound risk of PGD would be theoretically mitigated.

This theoretical concept of PGD risk assessment is shown in Table 2. The concept aims to standardize and objectify PGD risk by profiling the inflammatory status and/or phenotype of both the allograft and the recipient. An estimation of intraoperative risk is also included. The diagnostics required to assess recipient inflammatory risk reflect the need to profile the real-time pre-transplant inflammatory status of the recipient which may change according to factors such as worsening disease severity due to pulmonary inflammation and/or infection, At present, the only assay readily available in the preoperative setting is the neutrophil: lymphocyte ratio. Assays to determine cell free hemoglobin, the presence of lung-specific self-antigens and the immunophenotype have a current timeframe which would preclude their use in a clinical setting. Current innovations in diagnostic assays, such as developments in the real-time monitoring of hemolysis show that the relevant assays will become achievable (58).

**Table 2:** Concept of theoretical PGD risk compatibility matching on the basis of individualized recipient, surgical and specific allograft PGD risk factors utilizing future perspectives for advanced inflammatory diagnostics.

Recipient inflammatory risk	Surgical risk	Allograft LIRI risk	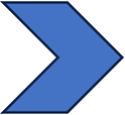	PGD risk
Low	Low	Low	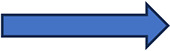	Low
Low	Low	High	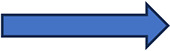	Intermediate
Low	High	Low	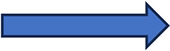	Intermediate
Low	High	High	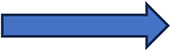	High
Intermediate	Low	Low	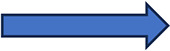	Intermediate
Intermediate	Low	High	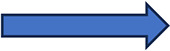	High
Intermediate	High	Low	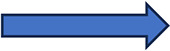	High
Intermediate	High	High	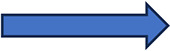	High
High	Low	Low	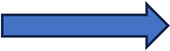	High
High	Low	High	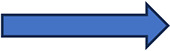	High
High	High	Low	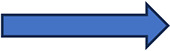	High
High	High	High	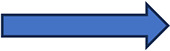	High

**Recipient Inflammatory Risk Low**=low inflammatory profile: low neutrophil: lymphocyte ratio, low risk immunophenotype, no antibodies present against lung-specific SAgs, no CFH present. **Recipient Inflammatory Risk Intermediate**=one of the following: pro-inflammatory profile, antibodies present against lung-specific SAgs, high neutrophil: lymphocyte ratio, high risk immunophenotype, CFH present. **Recipient Inflammatory Risk High**: more than one of the following: pro-inflammatory profile, antibodies present against lung-specific SAgs, high neutrophil: lymphocyte ratio, high risk immunophenotype, CFH present. **Surgical Risk Low**=no expected complications, low risk of significant transfusion of blood products, low risk of intraoperative ECC. **Surgical Risk High**=high surgical difficulty expected (including re-operation, adhesions), expected significant transfusion of blood products, expected use of ECC. **Allograft LIRI risk low**: diagnostics of EVLP perfusate or retrograde flush: low levels of inflammatory cytokines, no significant epithelial or endothelial injury present as diagnosed by Col-V, RAGE, cf mtDNA. **Allograft LIRI Risk High**: diagnostics of EVLP perfusate or retrograde flush: raised levels of inflammatory cytokines, epithelial and/or endothelial cell damage present as diagnosed by Col-V, RAGE, cf mtDNA, evidence for an ongoing activated innate immune response by NE DNA.

PGD, primary graft dysfunction; LIRI, lung ischemia reperfusion injury; SAgs, self-antigens; CFH, cell free haemoglobin; ECC, extra corporeal circulation; Col-V, collagen V; RAGE, receptor for advanced glycated end products; cf mtDNA, cell free mitochondrial DNA; NE DNA, neutrophil elastase DNA.

At present, EVLP is the only platform available which could enable the inflammatory profiling of lung allografts. However, in lung allografts with no clinical indications for EVLP, sampling the retrograde preservation flush may provide an alternative possibility to profile the inflammatory status of the endothelium in addition to BAL (if possible) as a way of profiling the epithelium in the period before procurement.

**Figure 2:**
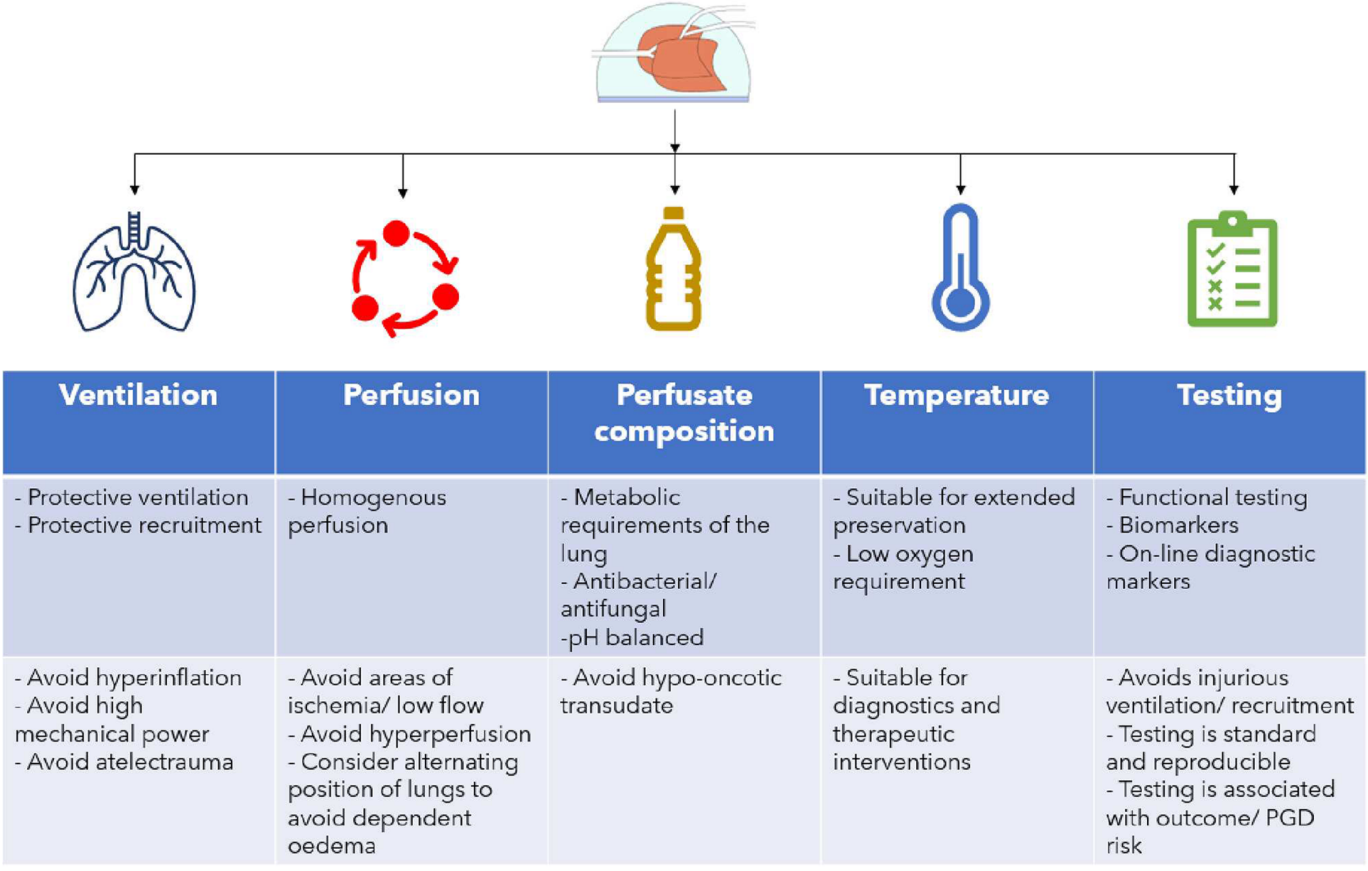
Optimising EVLP protocol for the goal of safe extended (>6 h) preservation. Ventilation, perfusion, perfusate, temperature and testing goals and considerations are shown.

Utilizing EVLP for advanced diagnostics of the lung allografts depends on the optimalization of the current EVLP protocols to enable safe extended preservation (>6 h) of lung allografts on EVLP (Figure 2) in order to facilitate the time required for the interpretation of diagnostics and eventual interventions. Adaptation of the current protocols to ensure safe extended EVLP must be based on an understanding of the altered lung dynamics and physiology encountered in the EVLP setting. Ventilation of lungs during EVLP must take into account the absence of a chest wall, heterogenicity in compliance of injured donor lungs and protect against further injury by minimizing the mechanical power conferred to the lungs during ventilation (59). It is reasonable to propose that a protective ventilation strategy for extended EVLP will be near-apneic. Perfusion protocols in extended EVLP must strive for homogeneous and adequate perfusion of the lungs which may require intermittently alternating their position to avoid hyperperfusion of dependent areas and the creation of a West's zone 1 (60) (alveolar pressure > pulmonary arterial and venous capillary pressure) in the upper lying regions of the lung reflecting limited blood flow and risk of ischemic injury to the pulmonary endothelium. The composition of the perfusate should ensure a colloid osmotic pressure that is adequate to prevent extravascular transudate and must take into account the metabolic demands and balance of the lung. In addition to these factors, the optimal temperature at which the lung is preserved allowing for accurate diagnostic testing and possible interventions must be determined.

### The concept of individualized PGD risk modification and targeted therapeutic interventions

A number of advanced therapeutic interventions have shown promise in the regeneration of lung function in injured allograft-lung transplant-PGD models, however, few have as yet been translated into the clinical setting. Two promising therapeutic interventions are the use of mesenchymal stromal (stem) cells (MSCs) and cytokine adsorption (61). MCSs exert an immunomodulatory effect on a wide range of inflammatory pathways and have been shown to have beneficial effects on injured lung allografts (62) and to help mitigate LIRI in experimental models of extended ischemia (63). Cytokine adsorption has been used clinically in a case series in an EVLP setting where a reduction of inflammatory mediators in donor lungs was shown, however, the study had insufficient power to demonstrate a positive effect on outcome (64). In a porcine damaged-lung allograft (ARDS) lung transplant model, cytokine adsorption helped restore lung function when instigated during organ preservation and continued post-transplant (65) therefore throughout the transplant continuum, while a clinical case series of post-operative cytokine adsorption in lung transplant recipients showed a trend to improved 1-year survival post-transplant (66). A recent small case series suggested a possible role for the perioperative use of cytokine adsorption in reducing NETosis (67) and PGD risk-reduction. By using the model of PGD risk assessment as individualized and specific to a certain transplant continuum it may be postulated that therapeutic interventions as standard in all donor lungs and/or all recipients will not confer improved outcome in all patients. Benefits of advanced therapeutic interventions may therefore be more specifically assessed in the specific targeted high-risk donor and/or recipient and/or surgical risk allocations.

**Figure 3:**
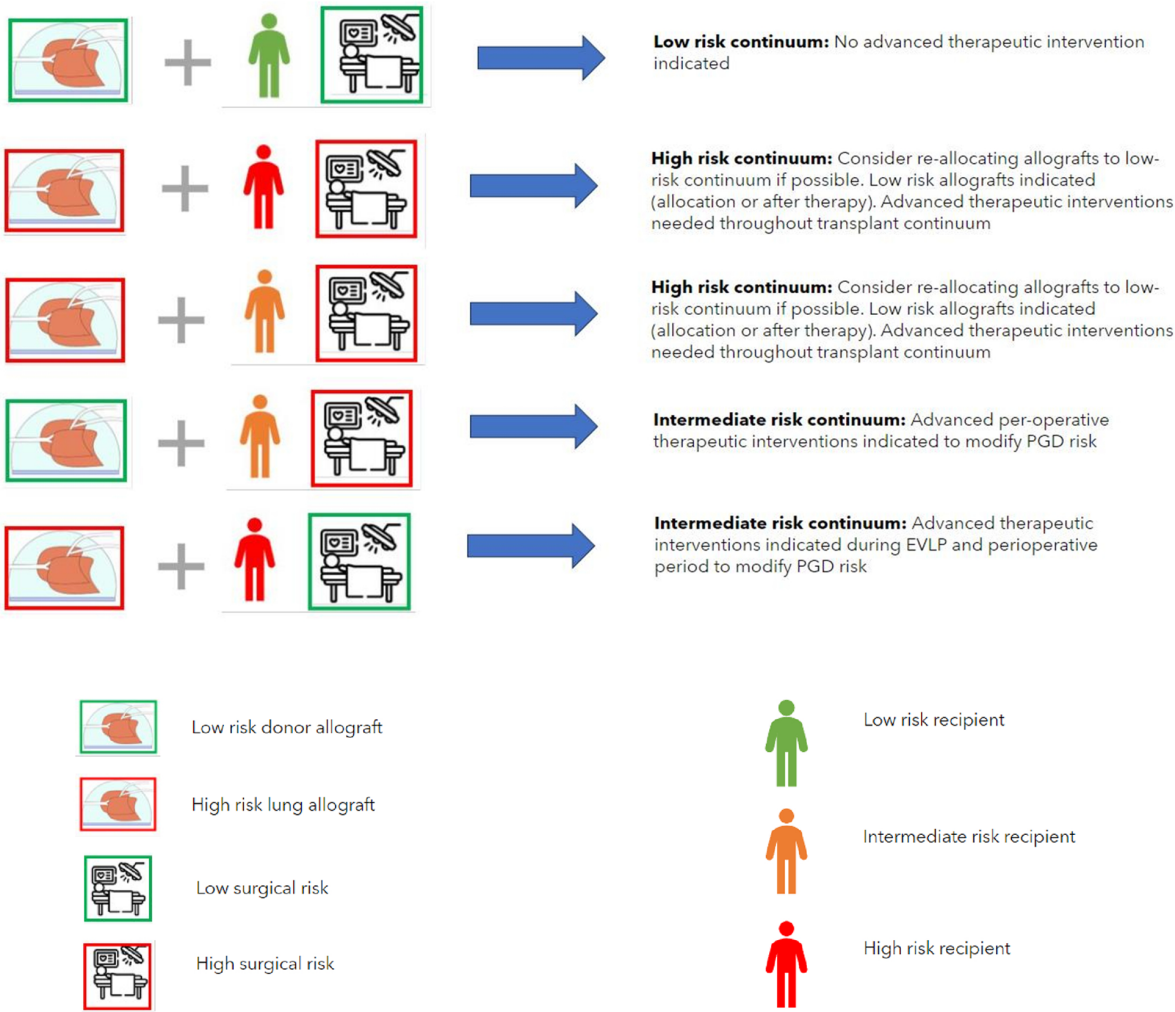
The concept of the allocation of donor lungs according to PGD compatibility. This requires safe extended preservation of donor lungs on EVLP, standardized (advanced inflammatory) diagnostics and testing of the donor lungs to designate them as low or high risk and standardized recipient inflammatory and immunophenotype assessment to designate the recipient as low, intermediate or high risk. Surgical risk for an individual recipient is estimated on the basis of the risk of use of perioperative extra corporeal circulation, blood loss and surgical difficulty. PDG risk modification may be performed as targeted advanced therapeutic interventions if high- or intermediate risk is present in donor-, recipient- or surgical factors (combinations shown reflect a low-risk continuum and examples of a high- or intermediate-risk continuum where advanced therapeutic options are indicated). **Key** (for definition of donor-, recipient- and surgical risk see legend Table 2).

The concept of PGD compatibility matching proposed in Table 2 may facilitate the future specific targeting of advanced therapeutic interventions according to individualized and contextualized PGD risk (Figure 3). It is reasonable to propose that if the total PGD risk is “intermediate” or “high” that therapeutic interventions on the lung allografts would be warranted and that subsequent interventions may be tailored to target the specific PGD risk. If the highest PGD risk is donor allograft-related, risk modulation therapy may be initiated during organ preservation in an EVLP setting. If the risk is predominantly recipient and/or intraoperative-risk related, then therapy may be reserved for the peri- and post-reperfusion period.

It may also be reasonable to propose that if the total PGD risk is low, that no advanced therapeutic interventions are warranted. Furthermore, in the case of a combination of a high-risk donor allograft, a high-risk recipient and a high interoperative risk the possibility of re-allocating the high-risk donor allograft to an individual recipient with a lower risk and/or lower interoperative risk may be considered, however this conceptual consideration may not be possible in the case of limited allograft offers for a patient who is at risk of imminent (terminal) respiratory failure. In this case the only option would be targeted PGD risk mitigation as described above. Whilst the combination of high-risk donor allografts and high-risk recipients is currently technically feasible with the knowledge that a high-grade inflammatory type PGD can be adequately supported post-operatively it must be kept in mind that salvage ECLS due to PGD following lung transplantation is associated with a longer duration of mechanical support post-operatively, longer hospital length of stay and poorer 1 and 3 year survival post-operatively (5). Furthermore, poorer longer-term outcomes of recipients who survive high-grade PGD in terms of risk of early CLAD development means that consideration should be given to a paradigm shift into the allograft allocation in the case of high-risk recipients. Avoiding the allocation of high-risk allografts in high-risk recipients is seems vital to an acceptable short, medium and long term outcome following lung transplant and may be most realistically achieved by targeted therapeutic interventions on specific donor lung allografts in an ex vivo setting with the aim of mitigating any allograft injury.

## Discussion

We present a hypothesis in which we argue that an individual lung transplant recipient's risk of inflammatory type PGD can potentially be identified and contextualized on the basis of the three variables of a specific transplant continuum: the recipient risk (immunophenotype and inflammatory status), the allograft risk of LIRI (based on peri mortal lung injury to the endothelium and epithelium and/or inflammation already present in the lung) and the intraoperative risk.

Translating this hypothetical concept into the clinical setting would require both innovation and standardization in the inflammatory diagnostic profiling of both the recipient and lung allografts, involving the ability to profile the recipient inflammatory status and immunophenotype, and the identification of donor lung specific markers for the presence of epithelial injury, endothelial injury and ongoing inflammation. We conceptualize that this inflammatory profiling and assessment may provide specific targets for (future) therapeutic strategies aimed at PGD risk modification. In the case of inflammation or injury in the donor lungs, immunomodulation strategies could be incorporated into an (extended) EVLP platform. In the case of a high-risk donor- recipient and intraoperative continuum these strategies may be required throughout the whole transplant continuum from the allograft preservation phase to the post-operative setting in the recipient.

Optimizing short- and long-term outcome following lung transplantation is strongly linked to the avoidance of high-grade inflammatory type PGD in the post-operative period. We argue that a strategy that approaches PGD risk reduction by assessing and potentially modifying specific aetiological risk factors present in an individual transplant continuum may lead improved lung transplant outcomes.

## Data Availability

The original contributions presented in the study are included in the article/Supplementary Material, further inquiries can be directed to the corresponding author.
